# Research Note: Complete genomic characterization and biological phenotyping of a genotype VI.2.1.1.2.2 pigeon-derived Newcastle disease virus strain HN25

**DOI:** 10.1016/j.psj.2026.108072

**Published:** 2026-10-31

**Authors:** Huiyou Xiao, Haoqian Wang, Luyao Song, Shuhui Sang, Chunmei Wang, Bingqian Zhou, Yang Zhang, Xinwei Wang

**Affiliations:** College of Veterinary Medicine, Henan Agricultural University, Zhengzhou Henan 450046, China

**Keywords:** Pigeon paramyxovirus type 1 (PPMV-1), Genotype VI, Fusion (F) protein, Hemagglutinin-neuraminidase (HN) protein

## Abstract

Pigeon paramyxovirus type 1 (PPMV-1) is a major cause of mortality in domestic and wild pigeons worldwide and has the potential to infect chickens, thereby posing a threat to the poultry industry. In this study, a PPMV-1 strain, designated HN25, was isolated and characterized. Its complete genome was sequenced and analyzed, and its biological and physicochemical properties were evaluated. Phylogenetic analysis classified HN25 into sub-genotype VI.2.1.1.2.2 (formerly VIk), which is the predominant sub-genotype currently circulating in China. The fusion (F) protein of HN25 contains the cleavage site motif ¹¹²RRQKR↓F¹¹⁷. The virulence indices—an EID₅₀ of 10⁷·⁶⁷/0.2 mL, a mean death time (MDT) of 55.2 h, and an intracerebral pathogenicity index (ICPI) of 1.75—indicate that HN25 is a virulent strain. Physicochemical characterization showed that HN25 is sensitive to chloroform and unstable under acidic (pH 6.0, 37°C, 1 h) and thermal (56°C, 30 min) conditions. Sequence alignment identified four mutations in the F protein within functional domains (P5S, P12S, C25Y, and D170N), with P5S, Q445K, A522T, and A549V being strain-specific relative to the consensus sequence. In the hemagglutinin-neuraminidase (HN) protein, only one mutation (I514V) was found within a neutralizing epitope, along with a rare non-conserved substitution (N440H). These findings provide baseline data on the genetic evolution and potential antigenic variation of the predominant PPMV-1 sub-genotype in China. The identified mutations offer candidate targets for future antigenicity studies and vaccine design, though experimental validation is required.

## Introduction

Newcastle disease (**ND**), caused by Newcastle disease virus (**NDV**), is a highly contagious avian disease imposing substantial economic burdens on the global poultry industry. Pigeon paramyxovirus type 1 (**PPMV-1**), a host-specific antigenic variant of NDV, is an enveloped, negative-sense RNA virus. Its surface glycoproteins F and HN are the principal immunogens inducing protective antibodies (Sheng et al., 2024; Ma et al., 2025). Although NDV exists as a single serotype, it exhibits remarkable genetic diversity. NDV is categorized into Class I and Class II, with the latter further divided into 20 genotypes based on the unified classification system proposed by Dimitrov et al. PPMV-1 predominantly belongs to Class II genotype VI, which remains one of the most epidemiologically prevalent lineages.

PPMV-1 was first identified in the Middle East in 1978 and introduced to mainland China in 1985, spreading rapidly thereafter. While all pigeon age groups are susceptible, with morbidity up to 80% and mortality exceeding 50% (Jin et al., 2025), China's massive meat pigeon industry (yielding ∼900 million squabs annually) faces significant management heterogeneity. In Henan Province, a central hub with 2.8–3.0 million breeding pairs, squabs are typically marketed at 22–35 days of age. During this late stage (especially after 18 days), waning maternal antibodies, suboptimal use of chicken NDV vaccines, and neglected biosecurity leave birds highly susceptible to field viruses, resulting in sporadic outbreaks.

In this study, we isolated a PPMV-1 strain (HN25) from a sporadic clinical case in Henan Province. By combining whole-genome sequencing with biological characterization, we analyzed its genetic evolution and potential antigenic variations to provide baseline data for targeted prevention and control.

## Materials and methods

The animal study was approved by the Scientific Ethics Committee of Henan Agricultural University, Henan, China. The study was conducted in accordance with the local legislation and institutional requirements.

### Virus isolation and identification

In 2025, a deceased pigeon exhibiting clinical signs of suspected Newcastle disease was submitted by an individual farmer from a local area in Henan Province, China. Liver and lung tissues were aseptically collected from the deceased pigeon. The tissues were homogenized, subjected to three freeze-thaw cycles, and centrifuged to collect the supernatant. The supernatant (0.2 mL) was inoculated into five 9- to 11-day-old specific-pathogen-free (SPF) chicken embryos. The allantoic fluid was collected and tested for Newcastle disease virus using hemagglutination (**HA**) and hemagglutination inhibition (**HI**) assays. Positive allantoic fluids were stored at −80°C for subsequent analysis.

### Whole-genome sequencing

Viral RNA was extracted from purified allantoic fluid using TRIGene reagent (Tiangen, Beijing, China) and reverse-transcribed using random hexamers. Sequencing libraries were prepared with the Nextera XT DNA Library Preparation Kit (Illumina, San Diego, CA, USA) and sequenced on the Illumina HiSeq platform (150 bp paired-end reads).

Raw reads were quality-controlled using FastQC (v0.11.2) and Trimmomatic (v0.36) to remove adapters and low-quality bases (Q < 20), yielding clean reads. *De novo* assembly was performed using SPAdes (v3.5.0), followed by gap filling (GapFiller v1.11) and sequence correction (PrInSeS-G v1.0.0). High sequencing depth was confirmed by the complete assembly of the 15,192 nt genome without gaps or ambiguous bases. The 5′ and 3′ termini were determined by mapping clean reads to the reference sequence (GenBank accession no. OR542543.1), and ORFs were predicted using Prokka (v1.10). The complete HN25 genome sequence has been deposited in GenBank under accession number QB174728.

### Virus titration and pathogenicity indices

The virus suspension was serially diluted 10-fold in sterile PBS (10⁻¹ to 10⁻⁹). Each dilution (0.2 mL per embryo) was inoculated into five SPF chicken embryos via the allantoic cavity. After incubation at 37°C, allantoic fluid from embryos that died between 24 and 120 h post-inoculation was collected for HA testing. The 50% embryo infectious dose (EID₅₀) was calculated using the Reed–Muench method based on HA positivity.

For MDT, the viral inoculum (0.1 mL/embryo) was administered to five SPF chicken embryos per dilution, and mortality was monitored at 37°C for 7 days. For ICPI, ten 1-day-old SPF chicks were inoculated intracerebrally with 0.05 mL of a 1:10 diluted viral solution, and clinical symptoms were scored daily for 8 days (0 = normal, 1 = sick, 2 = dead).

### Physicochemical characterization

***Chloroform sensitivity test.*** The virus suspension (10⁶ EID₅₀/mL) was divided into three 1-mL groups: 5% chloroform (50 µL), 10% chloroform (100 µL), and a saline control. After standing for 1 h and centrifugation (150 × g, 5 min), the aqueous phase was serially diluted (10⁻¹ to 10⁻⁸) and each dilution was inoculated into three 10-day-old SPF chicken embryos (0.2 mL/embryo). After incubation at 37°C for 72 h, allantoic fluid was collected for HA assay, and EID₅₀ was calculated. A > 1 log₁₀ reduction in EID₅₀ compared with the control indicated sensitivity. This experiment was performed only once; the three embryos per dilution were technical replicates, and no biological replicate was available. Therefore, results are presented descriptively without statistical testing.

***Acid and thermal stability tests.*** For acid stability, viral suspensions were adjusted to pH 4.0, 5.0, 5.5, 6.0, 6.5, and 7.0, and incubated at 37°C for 1 h. An unadjusted viral suspension incubated under the same conditions served as the positive control. After incubation, samples were neutralized to pH 7.2–7.4 and assayed for HA titer (log₂). For thermal stability, viral suspensions were incubated at 56°C for 0, 15, 30 min, 1, 2, 3, and 4 h, and the reaction was terminated in an ice bath. An untreated viral suspension stored at 4°C served as the positive control, and HA titers were determined. Both experiments were independently repeated three times (n = 3 biological replicates). Within each independent experiment, one sample was used per group and measured three times, with each HA measurement performed in one well. The three technical replicates were averaged to obtain one representative value per independent experiment.

### Phylogenetic analysis and bioinformatics predictions

A total of 43 complete F and HN gene sequences were retrieved from GenBank. Sequences were aligned using ClustalW in MEGA 7.0 with pairwise deletion. Phylogenetic trees were constructed using the neighbor-joining method based on the Maximum Composite Likelihood model with 1,000 bootstrap replicates. La Sota (AF077761.1) was used as the outgroup, and genotype assignment followed Dimitrov et al. Accession numbers are listed in Fig. 1. Signal peptides, transmembrane domains, and antigenic epitopes were predicted using SignalP 6.0, TMHMM 2.0, and IEDB, respectively; homology modeling used SWISS-MODEL. Data were visualized using Origin 2024.

**Fig. 1:**
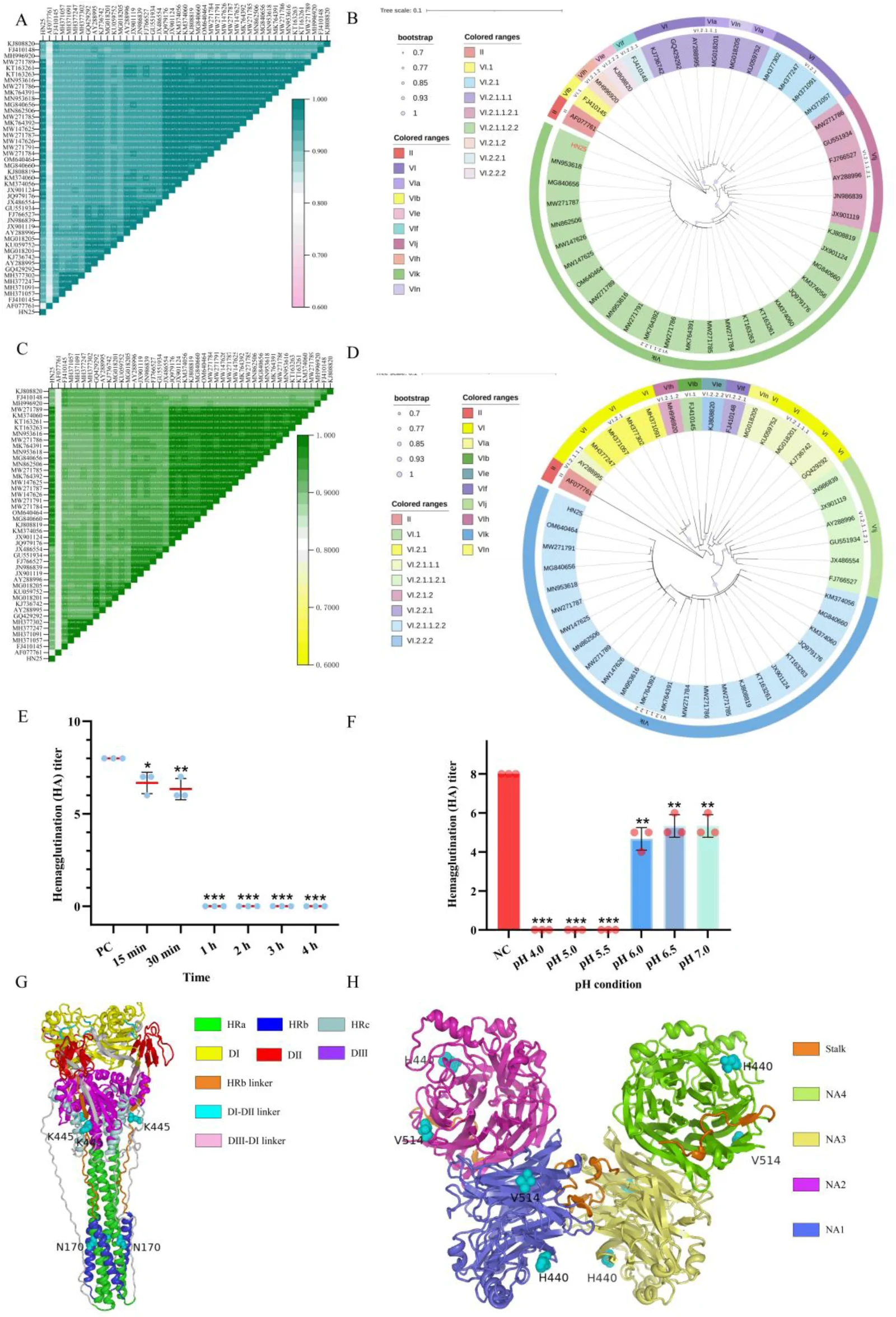
Physicochemical Characteristics of PPMV-1 and Genetic Evolution Analysis of Its F and HN Genes. (A) Triangular heatmap showing nucleotide sequence homology of the F gene. Color intensity indicates the percentage of sequence identity. (B) Phylogenetic tree of F gene nucleotide sequences constructed using the neighbor joining method with 1,000 bootstrap replicates (the target virus strain is indicated in red). (C) Triangular heatmap showing nucleotide sequence homology of the HN gene. Color intensity indicates the percentage of sequence identity. (D) Phylogenetic tree of HN gene nucleotide sequences constructed using the neighbor joining method with 1,000 bootstrap replicates (the target virus strain is indicated in red). (E) Thermal stability of NDV at 56 °C. Asterisks indicate significant differences compared with the untreated positive control (PC) at the same time point, as determined by one-way ANOVA followed by Dunnett's multiple comparison test (*p < 0.05, **p < 0.01, ***p < 0.001). (F) Effect of acid on the hemagglutination activity of NDV. Asterisks indicate significant differences compared with the PC, as determined by one-way ANOVA followed by Dunnett's multiple comparison test (*p < 0.05, **p < 0.01, ***p < 0.001). (G) Structural modeling of the F protein. (H) Structural modeling of the HN protein.

### Statistical analysis

Analyses were performed using GraphPad Prism 10.0. Acid and thermal stability data are presented as mean ± SD from three independent experiments (n = 3). Normality and variance homogeneity were assessed via Shapiro-Wilk and Levene’s tests, respectively. One-way ANOVA with Dunnett’s post hoc test (or Kruskal-Wallis with Dunn’s test for non-parametric data) was used to compare treatments with the control. A P-value < 0.05 was considered significant. The single-run chloroform test is presented descriptively without statistical testing.

## Results and discussion

A PPMV-1 strain, designated HN25, was successfully isolated. Its complete genome is 15,192 nt in length, conforming to the "rule of six." Homology analysis revealed that the *F* gene of HN25 shares 99.1%–99.6% nucleotide identity with genotype VI strains, while the *HN* gene shares 98.2%–99.6% identity. Both genes shared the lowest nucleotide identity with the genotype II vaccine strain La Sota (92.2% and 92.6%, respectively), indicating significant genetic divergence between HN25 and genotype II strains (Fig. 1A and C). Phylogenetic analysis based on the *F* and *HN* gene sequences showed that HN25 clustered with recent isolates from eastern and central China (PPMV-1/Gansu/02/2020 and pigeon/Sichuan/1433/2019). All these strains belong to sub-genotype VI.2.1.1.2.2 (formerly VIk), which is currently the predominant sub-genotype circulating in China (Jin et al., 2025) (Fig. 1B and D). Sequence analysis of the cleavage site motif revealed that the F protein of HN25 contains the sequence ¹¹²RRQKR↓F¹¹⁷, which is characteristic of virulent PPMV-1 strains and differs markedly from the La Sota strain (¹¹²G/E-K/R-Q-G/E-R-L¹¹⁷). Further biological evaluation of HN25 showed that the strain is sensitive to organic solvents (inactivated by chloroform), acid-labile (after 1 h at pH 6.0, the HA titer decreased from 8.0 to 4.7 log₂, with a relative activity of 10.1%), and thermolabile (HA activity completely lost within 1 h at 56°C). The EID₅₀ was 10⁷·⁶⁷/0.2 mL, the MDT was 55.2 h, and the ICPI was 1.75. These indices meet the World Organisation for Animal Health **(WOAH**) criteria for virulent strains, confirming that HN25 is a virulent PPMV-1 strain.

Furthermore, systematic alignment of the F and HN protein amino acid sequences between HN25 and reference strains revealed multiple mutations in both proteins. Four mutations in the F protein are located within functional domains (P5S, P12S, C25Y, and D170N), and four strain-specific mutations (present only in HN25) were identified (P5S, Q445K, A522T, and A549V). These mutations are distributed across key functional domains, including the signal peptide, heptad repeat A (**HRa**), HRB linker, transmembrane (**TM**) domain, and cytoplasmic tail (**CT**). The signal peptide is responsible for targeting the F protein to the endoplasmic reticulum and regulating fusion activity (Ma et al., 2025). NetPhos 3.1 predicted P5S to be a high-confidence phosphorylation site. P12S results in a proline-to‑serine substitution, and C25Y disrupts the only cysteine residue in this region, suggesting that these three mutations may affect F protein synthesis by altering signal peptide processing or folding and trafficking. Although phosphorylation of the PPMV-1 P protein has been shown to affect viral transcription and replication (Qiu et al., 2016), the F and P proteins have distinct functions. Whether residue S5 of the F protein is actually phosphorylated and whether it exerts similar functional consequences remains unknown.

D170N is located in the α-helix of HRa, resulting in the loss of a negative charge at this position. Because HRa and HRb assemble into a six-helix bundle through electrostatic interactions (Swanson et al., 2010), D170N may affect the stability of the six-helix bundle. Q445K is located in the HRB linker, a region where key residues can regulate fusion capacity (Liu et al., 2021; Ma et al., 2025); therefore, Q445K may disrupt the local electrostatic environment and affect the fusion conformation. A522T is located in the TM domain and may alter the hydrophobic environment, thereby affecting membrane anchoring (Ma et al., 2025). A549V is located in the CT; although mutations in this region have been reported to enhance fusion and pathogenicity (Samal et al., 2013), previous studies did not specifically target the A549 residue. In summary, these mutations constitute the genetic characteristics of HN25 and suggest potential impacts on F protein synthesis, fusion, membrane anchoring, and viral replication and pathogenicity. However, these functional consequences remain hypotheses and require validation through site-directed mutagenesis, fusion activity assays, reverse genetics, and in vivo experiments.

Compared with the F protein, variation in the neutralizing epitopes of the HN protein is extremely limited. Among the nine epitope regions analyzed, only I514V was detected within the 513–521 epitope, whereas all other known linear epitope residues matched the consensus sequence exactly. In addition, the HN protein of HN25 carries a rare, non-conserved substitution, N440H. I514V is located within a neutralizing epitope. Previous studies have reported that this substitution in the HN protein of virulent NDV strains enhances hemagglutination (HA) and neuraminidase (**NA**) activities and improves viral attachment to host cells (Babaeimarzangou et al., 2023); however, its antigenic consequences in HN25 require serological and reverse genetics validation.

N440H is located at the C-terminal region of the HN globular head. Most reference strains carry S or N at this position, whereas HN25 carries H, which may form an HH pair with the adjacent H439. Histidine protonation can act as a pH-sensitive charge switch (Schönichen et al., 2013). In Sendai virus and human parainfluenza virus type 3 (hPIV3), a histidine switch in the HN protein contributes to fusion promotion (Krishnan et al., 2009). However, this evidence originates from other paramyxoviruses, and whether a similar mechanism exists in NDV HN remains unclear. Therefore, we only hypothesize that N440H may affect HN conformational stability or fusion-related functions by altering local charge or pH sensitivity. This hypothesis requires validation through site-directed mutagenesis and functional assays.

This study has limitations. The chloroform sensitivity test was performed only once without biological replicates, so the results are descriptive. Additionally, because HN25 was isolated from a sporadic clinical case submitted by an individual farmer, detailed flock-level data (e.g., morbidity, mortality, and vaccination status) were unavailable. However, the occurrence of this case aligns with current field conditions: as maternal antibodies wane in late-stage squabs, exposure to field viruses often leads to clinical disease, particularly in small-scale farms with suboptimal vaccination and biosecurity. Although HN25 belongs to the predominant sub-genotype VI.2.1.1.2.2, the unique genetic variations identified in this field isolate underscore the ongoing evolution of PPMV-1 and highlight the need for enhanced epidemiological surveillance and tailored vaccination strategies in the region (Table 1).

**Table 1:** Amino acid mutations in the functional domains of the f protein and the neutralizing epitopes of the HN protein of strain HN25.

Functional domain / epitope	Consensus*	HN25	LaSota
F protein signal peptide (1–31)	MDSKPHIRIPAPPTLITRITLVLSCICSTSS	P5S, P12S, C25Y	D2G, K4R, H6S, H7T, R8K, I9N, P13M, T14M, I16T, T17I, I19V, T20A, S28P, T29A, S30N
F protein fusion peptide (117–141)	FIGAIIGSVALGVATSAQITAAAAL	–	F117L, S124G, S132A
F protein heptad repeat region A (143–185)	QANQNAANILRLKESIAATNEAVHEVTDGLSQLAVAIGKMQQF	D170N	N145K
F protein heptad repeat region B (268–299)	LITGNPILYDSQTQLLGIQVNLPSVGNLNNMR	–	N288T
HN epitope 193–201	LSGCIDHSH	–	I197R
HN epitope 263	K	–	N
HN epitope 287	D	–	–
HN epitope 321	K	–	–
HN epitope 332–333	GK	–	–
HN epitope 346–353	DGQEYQIR	–	G347E, E349D
HN epitope 356	K	–	–
HN epitope 494	D	–	G
HN epitope 513–521	RVTRVSSSS	I514V	V514I
HN epitope 569	E	–	D

*Consensus sequence derived from alignment of reference strains.

## Ethics declaration

### Animal subject

This study was conducted in accordance with the ARRIVE (Animal Research: Reporting of In Vivo Experiments) guidelines.

This study was approved by the Scientific Ethics Committee of Henan Agricultural University.

### Author contributions

Huiyou Xiao conceived and designed the study, performed the experiments, collected and analyzed the data, and drafted the manuscript. Haoqian Wang performed the experiments, collected and analyzed the data, and drafted the manuscript. Luyao Song conceived the study, performed formal analysis, curated the data, and prepared the figures. Shuhui Sang conceived the study, developed the methodology, and administered the project. Chunmei Wang supervised the study, validated the results, and performed the experiments. Bingqian Zhou supervised the study, validated the results, and contributed to the conception of the study. Yang Zhang supervised the study, performed formal analysis, and developed the methodology. Xinwei Wang conceived and designed the study, developed the methodology, supervised the study, and critically revised the manuscript. All authors have approved the final version and agree to be accountable for all aspects of the work.

## Disclosures

The authors declare that they have no known competing financial interests or personal relationships that could have appeared to influence the work reported in this paper.
